# Monitoring subnational regional inequalities in health: measurement approaches and challenges

**DOI:** 10.1186/s12939-016-0307-y

**Published:** 2016-01-28

**Authors:** Ahmad Reza Hosseinpoor, Nicole Bergen, Aluisio J. D. Barros, Kerry L. M. Wong, Ties Boerma, Cesar G. Victora

**Affiliations:** Department of Information, Evidence and Research, World Health Organization, Geneva, Switzerland; International Center for Equity in Health, Federal University of Pelotas, Pelotas, Brazil

**Keywords:** Area-based unit of measure, Health inequality, Measures of inequality, Monitoring, Regional inequality, Summary measures

## Abstract

**Background:**

Monitoring inequalities based on subnational regions is a useful practice to unmask geographical differences in health, and deploy targeted, equity-oriented interventions. Our objective is to describe, compare and contrast current methods of measuring subnational regional inequality. We apply a selection of summary measures to empirical data from four low- or middle-income countries to highlight the characteristics and overall performance of the different measures.

**Methods:**

We use data from Demographic and Health Surveys conducted in Bangladesh, Egypt, Ghana and Zimbabwe to calculate subnational regional inequality estimates for reproductive, maternal, newborn, and child health services generated from 11 summary measures: pairwise measures included high to low absolute difference, high to low relative difference, and high to low ratio; complex measures included population attributable risk, weighted variance, absolute weighted mean difference from overall mean, index of dissimilarity, Theil index, population attributable risk percentage, coefficient of variation, and relative weighted mean difference from overall mean. Four of these summary measures (high to low absolute difference, high to low ratio, absolute weighted mean difference from overall mean, and relative weighted mean difference from overall mean) were selected to compare their performance in measuring trend over time in inequality for one health indicator.

**Results:**

Overall, the 11 different measures were more remarkable for their similarities than for their differences. Pairwise measures tended to support the same conclusions as complex summary measures–that is, by identifying same best and worst coverage indicators in each country and indicating similar time trends. Complex measures may be useful to illustrate more nuanced results in countries with a great number of subnational regions.

**Conclusions:**

When pairwise and complex measures lead to the same conclusions about the state of subnational regional inequality, pairwise measures may be sufficient for reporting inequality. In cases where complex measures are required, mean difference from mean measures can be easily communicated to non-technical audiences.

## Background

Subnational regional health inequality is defined as the variability in a given health indicator between populations living in geographically-defined regions (provinces, states, etc.). The rationale for measuring subnational regional-based inequality derives from the assumption that populations of a region share similar conditions that directly or indirectly affect health. These may include health system inputs and processes, the availability of other services (e.g. education), local infrastructure, climate, environmental contaminants, proximity to facilities, or the acceptability of services (e.g. local culture). Furthermore, regions are the administrative units linked to resource allocation. Thus, monitoring health inequalities between regions can generate important evidence and support for targeting of health programs and policies, especially when disparities are substantial [1]. We note that this is a distinct concept from measuring total inequality within a population, which is a univariate measure of the distribution of health within a population.

As a starting point, disaggregated data from regions should be presented for visual inspection, but may be cumbersome to interpret when several health indicators are presented over a number of years for multiple regions [2]. Moreover, the interpretation of time trends becomes further complicated when the relative size of regions varies over time. Building on disaggregated data, measuring and describing regional inequality can be done in a number of ways using summary measures.

Summary measures of inequality condense disaggregated data into concise outputs, and can thus be used to show trends and make comparisons. The selection of appropriate regional summary measures entails a few considerations [2–4]. First, measures may demonstrate absolute inequality (i.e., the absolute magnitude of difference, retaining the unit of measure of the health indicator) or relative inequality (i.e., proportional differences that do not retain the unit of measure). Several summary measures of inequality have both absolute and relative versions. Second, measures that facilitate pairwise comparisons between two regions can be distinguished from measures that simultaneously take all regions into account. Third, measures of inequality may be based on weighted or unweighted data, according to whether or not the population size in each region is taken into account. Finally, the choice of the reference point should be justified based on the intended purposes of the analysis. Reference points are commonly defined as the level of health in the best performing region, health in a region with special significance (such as the capital region), the overall mean health of all regions (i.e. national average), or a predetermined standard level of health. The choice of such a point has important implications when interpreting inequality measures.

Based on these considerations, each type of summary measure has implicit advantages and disadvantages, and some are more intuitive than others. A review of the published literature identified four main categories of summary measures applicable to regional inequality (Panel 1).

The overall objective of the paper is to describe, compare and contrast current methods of measuring subnational regional inequality. We use empirical data from four countries to calculate inequality estimates for reproductive, maternal, newborn, and child health services generated from 11 summary measures. We identify criteria for determining the most robust, simplest, and consistent set of inequality measures, and discuss the implications for reporting subnational regional inequality.

### Panel 1. Typology of summary measures of regional inequalities

#### Pairwise measures

The most basic measures of subnational regional inequality include pairwise measures such as differences and ratios. For example, the mean level (or a proportion or rate) of a health indicator in region A may be compared to the mean in region B, or mean in region A may be compared to the overall national mean. Because pairwise measures are straightforward and comprehensible, they are ideal when only two areas are being compared. However, these cannot be used to generate a single summary estimate for multiple areas. In this case, pairwise comparisons are still possible but a reference group must be defined, as was done to compare infant mortality rates in the five regions of Brazil. The rate ratio between the region with the highest mortality (the Northeast) and the region with the lowest (the South) decreased from 2.6 in 1990 to 2.2 in 2007; the absolute difference between these two regions decreased from 47.1 deaths per 1000 live births in 1990 to 15.3 deaths per 1000 live births in 2007 [5].

#### Measures of disproportionality

Measures of disproportionality look at the ‘share of health’ in a population that is experienced by a given share of a subpopulation. (The share of health may encompass health outcomes, health services or other health indicators.) For instance, the index of dissimilarity shows the proportion or number of people who would have to move to a different region to achieve a uniform distribution of health across a country [6]. It may be expressed in absolute (actual number of individuals) or relative (proportion of the population) scales. The relative version of the index was calculated for four maternal health service coverage indicators across 94 counties/cities in China. The smallest inequalities were in hospital delivery rate (index of dissimilarity = 6 %, meaning that 6 % of the population would need to be redistributed to achieve a uniform distribution of coverage across regions). The other indicators had indices of dissimilarity of 11 % (for examinations rate), 18 % (for more than four postnatal examinations), and 21 % (for more than four prenatal examinations). These analyses motivated the integration of subsequent maternal health programmes and policies with a regional focus [7].

The Theil index is also based on the concept of disproportionality, measuring relative inequality. It takes into account the proportion of the population in each region and the prevalence ratios of the health indicators in each region to the national mean value. The Theil index has a minimum calculated value of 0 (no regional inequality); as relative inequality increases, the value becomes larger, with no upper bound. If a populous region has a much higher level of health than the national average the Theil index will be inflated, indicating greater inequality. Theil index values may be scaled (for example, uniformly multiplied by 1000) to facilitate interpretation. For example, the Theil index was used to measure inequalities in the availability of health workers among 22 provinces in China. It showed greater inequality in per-head availability of nurses (Theil index value = 0.067) than doctors (0.043); this was also observed when inequality was analyzed at county level, with a higher Theil index value for nurse (0.408) than doctor availability (0.235) [8].

#### Measures of impact

When applied to subnational regional inequalities, population attributable risk shows the total health improvement expected at national level if all regions had the same level of health as the reference group (often defined as the best performing region). The measure takes into account the population size. A relative version, population attributable risk percentage, shows the proportional improvement possible if all regions attained the same level of health as the best performing region. Absolute and relative versions of population attributable risk have been used to show that the number of smokers in Montreal could be reduced by 176,869 people (population attributable risk) or 55 % (population attributable risk percentage) if all city neighborhoods matched the one with the lowest smoking prevalence [9].

#### Measures of variance

Measures based on the principle of variance aim to show how widely spread are the levels of a health indicator in multiple geographical areas. Variance is the sum of the squared differences between the level of health in each region and the overall level, divided by the number of regions. It provides an absolute estimate of inequality, which may be unweighted or weighted. The weighted (or between-group) variance approach was applied to road traffic injury mortality across 22 cities (or counties) in Taiwan. Differences between mortality in each city and the overall mean were squared and multiplied by the city population size; the resulting value, divided by the national population produced the between group variance, which decreased from 179 in 1997 to 49 in 2008 [10].

The standard deviation, or square root of unweighted variance, was used to track regional fertility inequalities in rural Iran. The standard deviation of the percentage of births attended by unskilled personnel fell from 15.3 to 10.9 percentage points between 1996–2000 and 2001–2005, indicating decreased inequality [11].

Coefficient of variation is a relative version of standard deviation, expressed as a percentage of the overall mean [12]. Being a relative measure, it allows comparison of the magnitude of inequalities for different health indicators–even those that have different units of measurement–which is not possible with the variance or standard deviation approaches. Additionally, coefficient of variation takes into account the overall mean, allowing comparisons over time when the overall mean may have changed. A study in 17 countries from the Middle East and North Africa from 1980 to 1994 showed that while the mean under-five mortality rate in the region decreased from 144.5 to 62.4 deaths per 1000 live births, the coefficient of variation increased from 28.8 % in 1980 to a maximum of 52.3 % in 1992 [13].

Measures of mean differences from mean show how each region differs from a reference point. The measure expressed as absolute or relative inequality, and may be weighted or unweighted. Depending on the purposes of the comparison, reference points may include the mean level of the whole population (a measure referred to as ‘mean difference from overall mean’), the level of health in the best-performing region (a measure referred to as ‘mean difference from best’), or a predetermined target level of health [2]. One specific formulation is known as the index of disparity, calculated as the average of the absolute differences between the levels in each region and the overall mean, divided by the overall mean and expressed as a percentage [6, 14]. The index of disparity was used to summarize regional inequalities in under-five death rates in Iran over 1993–2009, and spanned from 24.4 % in 1995 to 17.6 % in 2007 [15].

## Methods

### Data sources

Data about four reproductive, maternal, newborn, and child health service indicators were used to generate estimates of regional inequality within four countries. Data were obtained from Demographic and Health Surveys (DHS) conducted over 1996–2007 (Bangladesh), 1995–2008 (Egypt), 1998–2008 (Ghana), and 1999–2010 (Zimbabwe); data for Bangladesh and Egypt are obtained from four survey rounds, and Ghana and Zimbabwe each reported data from three survey rounds. DHS is a large-scale, nationally-representative household survey program that routinely collects and disseminates data about a range of health and demographic indicators from over 90 low and middle-income countries [16]. These countries were selected for inclusion because they represent multiple World Health Organization regions, and each reported data for at least three time points in the period 1995–2010 for a constant number of subnational regions (thus facilitating tracking of time trends in regional inequalities). Additionally, comparisons could be made between pairs of countries with the same number of regions, as Bangladesh and Egypt each reported on six regions, and Ghana and Zimbabwe, ten.

Four reproductive, maternal, newborn, and child health indicators were studied: demand for family planning satisfied, antenatal care (at least one visit with a skilled health provider), births attended by skilled health personnel, and measles immunization coverage among 1-year-olds. These four indicators were available for all four countries and from three or four survey rounds. A detailed description of the indicator numerators and denominators can be found on the World Health Organization Global Health Observatory Health Equity Monitor [17].

### Analysis

Eleven summary measures were applied to demonstrate subnational regional inequality (Panel 2). Absolute measures included high to low absolute difference, population attributable risk, weighted variance, and absolute weighted mean difference from overall mean; relative measures included high to low relative difference, high to low ratio, index of dissimilarity, Theil index, population attributable risk percentage, coefficient of variation, and relative weighted mean difference from overall mean. The characteristics of these measures are shown in Table 1. The 11 summary measures are compared across the four indicators within each country. Next, four summary measures are selected to compare trends over time for one indicator (demand for family planning satisfied) within each country. The family planning indicator was selected because it demonstrated variable patterns over time, and thus permitted comparisons by summary measure.

**Table 1:** Characteristics of selected summary measures of within-country regional inequality

	Summary measure	Category of measure (subgroups included)	Unweighted or weighted	Reference group
Absolute measures of inequality	High to low absolute difference	Pairwise (extreme subgroups)	Unweighted	Best region
	Population attributable risk	Impact (all groups)	Weighted	Best region
	Weighted variance	Variance (all groups)	Weighted	Overall mean
	Absolute weighted mean difference from overall mean	Variance (all groups)	Weighted	Overall mean
Relative measures of inequality	High to low relative difference	Pairwise (extreme groups)	Unweighted	Best region
	High to low ratio	Pairwise (extreme groups)	Unweighted	Best region
	Index of dissimilarity	Disproportionality (all groups)	Weighted	Overall mean
	Theil index	Disproportionality (all groups)	Weighted	Overall mean
	Population attributable risk percentage	Impact (all groups)	Weighted	Best region
	Coefficient of variation	Variance (all groups)	Weighted	Overall mean
	Relative weighted mean difference from overall mean	Variance (all groups)	Weighted	Overall mean

### Panel 2. Summary measures for regional inequality: formulae and application

Any of the summary measures for regional inequality detailed in the main text can be calculated if data are available about the national and region levels of the health indicator, and the corresponding weighted sample size (where the data source is household surveys) and population share. The formulae and application for a selection of 11 regional inequality summary measures are demonstrated using data about coverage of births attended by skilled health personnel in six regions of Bangladesh.

#### Births attended by skilled health personnel, Bangladesh, DHS 2007

a. National coverage and coverage by subnational region

**Table:** 

National coverage weighted sample size (share of total population)	Coverage by subnational region weighted sample size (share of total population)					
	Barisal	Chittagong	Dhaka	Khulna	Rajshahi	Sylhet
17.9 %	13.3 %	18.4 %	19.6 %	26.7 %	15.4 %	10.9 %
6058 (1.00)	383 (0.06)	1337 (0.22)	1908 (0.31)	578 (0.10)	1306 (0.22)	547 (0.09)

b. Application of summary measures

**Table:** 

Summary measure	Description	Formula^a^	Sample calculation
Absolute measures of inequality			
High to low absolute difference	Shows the absolute difference in health between the best- and worst-performing regions	*r*_(*high*)_ − *r*_(*low*)_	26.7 − 10.9 = 15.8
Population attributable risk	Shows the total health improvement possible in the population if all regions had the same level of health as the reference point (such as national average).	*r*_(*high*)_ − *r*	26.7 − 17.9 = 8.8
Weighted variance	Shows the sum of the differences between the level of health in each region (weighted) and the overall level squared, divided by the number of regions.	$\sum \frac{po{p}_{(i)}\times {\left({r}_{(i)}-r\right)}^2}{pop}$	383 × (13.3 − 17.9)^2^+ 1337 × (18.4 − 17.9)^2^ + 1908 × (19.6 − 17.9)^2^ + 578 × (26.7 − 17.9)^2^ + 1306 × (15.4 − 17.9)^2^ + 547 × (10.9 − 17.9)^2^)/6058 = 15.5
Absolute weighted mean difference from overall mean	Shows the difference of health in each region (weighted) from a reference point	$\sum \frac{po{p}_{(i)}\times \left|{r}_{(i)}-r\right|}{pop}$	383 × |13.3 − 17.9| + 1337 × |18.4 − 17.9| + 1908 × |19.6 − 17.9| + 578 × |26.7 − 17.9| + 1306 × |15.4 − 17.9| + 547 × |10.9 − 17.9|)/6058 = 3.0
Relative measures of inequality			
High to low relative difference	Shows the relative difference in health between the best- and worst-performing regions as a percentage of the level of health in the best-performing region	$\frac{r_{(high)}-{r}_{(low)}}{r_{(high)}}\times 100$	(26.7 − 10.9)/26.7 × 100 = 59.2
High to low relative ratio	Shows the ratio in health between the best- and worst-performing regions	$\frac{r_{(high)}}{r_{(low)}}$	26.7/10.9 = 2.4
Index of dissimilarity	Shows the proportion of people that would have to move to a different region to achieve a uniform distribution of health across a population	$0.5\times \sum \left|\frac{r_{(i)}}{r}\times \frac{po{p}_{(i)}}{pop}-\frac{po{p}_{(i)}}{pop}\right|\times 100$	0.5 × (|(13.3/17.9) × (383/6058) − (383/6058)| + |(18.4/17.9) × (1337/6058) − (1337/6058)| + |(19.6/17.9) × (1908/6058) − (1908/6058)| + |(26.7/17.9) × (578/6058) − (578/6058)| + |(15.4/17.9) × (1306/6058) − (1306/6058)| + |(10.9/17.9) × (547/6058) − (547/6058)|) × 100 = 8.2
Theil index	Shows relative inequality, taking into account the proportion of the population in each region and the ratio of the health indicator prevalence in each region to the national mean health indicator prevalence	$\sum \frac{po{p}_{(i)}}{pop}\times \frac{r_{(i)}}{r}\times \ln \frac{r_{(i)}}{r}\times 1000$	(383/6058 × 13.3/17.9 × ln(13.3/17.9)) + (1337/6058 × 18.4/17.9 × ln(18.4/17.9)) + (1908/6058 × 19.6/17.9 × ln(19.6/17.9)) + (578/6058 × 26.7/17.9 × ln(26.7/17.9)) + (1306/6058 × 15.4/17.9 × ln(15.4/17.9)) + (547/6058 × 10.9/17.9 × ln(10.9/17.9)) × 1000 = 24.1
Population attributable risk percentage	Shows the proportional improvement possible if all regions attained the same level of health as the reference point	$\frac{PAR}{r}\times 100$	8.8 /17.9 × 100 = 49.2
Coefficient of variation	Shows the standard deviation as a percentage of the overall mean (i.e. the square root of weighted variance divided by the overall mean)	$\frac{\sqrt{WV}}{r}\times 100$	(√ 15.5)/17.9 × 100 = 22.0
Relative weighted mean difference from overall mean	Shows the amount of deviation from the overall mean (weighted by region) as a percentage of the overall mean level of health	$\frac{WMDM}{r}\times 100$	3.0/17.9 × 100 = 16.5

^a^r denotes overall national coverage; r_(low)_ denotes coverage of the worst-performing region, and r_(high)_ denotes coverage of the best-performing region; r_(i)_ denotes coverage within a specified region i; pop denotes the overall weighted sample size; pop_(i)_ denotes the weighted sample size within a specified region i; n denotes the number of regions

## Results

### Disaggregated data

Regionally-disaggregated data and national coverage levels of four selected health services are presented for Bangladesh, Egypt, Ghana, and Zimbabwe (Table 2). These data were used to calculate the 11 measures of inequality for the four health indicators.

**Table 2:** Four reproductive, maternal, newborn, and child health intervention indicators: coverage at national and subnational levels in Bangladesh, Egypt, Ghana, and Zimbabwe, DHS 2007–2010

		National coverage	Coverage, by subnational region (share of total population)									
Bangladesh, 2007			Barisal	Chittagong	Dhaka	Khulna	Rajshahi	Sylhet				
	Demand for family planning satisfied (%)	92.3	88.2 (0.06)	88.8 (0.15)	93.5 (0.31)	95.6 (0.14)	93.7 (0.30)	81.7 (0.04)	–	–	–	–
	Antenatal care coverage (at least one visit) (%)	51.7	43.7 (0.06)	52.4 (0.21)	48.2 (0.32)	62.6 (0.10)	54.9 (0.23)	46.9 (0.08)	–	–	–	–
	Births attended by skilled health personnel (%)	17.9	13.3 (0.06)	18.4 (0.22)	19.6 (0.31)	26.7 (0.10)	15.4 (0.22)	10.9 (0.09)	–	–	–	–
	Measles immunization coverage (%)	83.1	90.2 (0.06)	79.6 (0.24)	83.3 (0.31)	89.6 (0.08)	86.1 (0.22)	73.1 (0.08)	–	–	–	–
Egypt, 2008			Urban Governorates	Lower Egypt- Urban	Lower Egypt- Rural	Upper Egypt- Urban	Upper Egypt- Rural	Frontier Governorate				
	Demand for family planning satisfied (%)	87.0	91.7 (0.18)	91.1 (0.12)	89.3 (0.36)	88.8 (0.11)	76.1 (0.22)	84.1 (0.01)	–	–	–	–
	Antenatal care coverage (at least one visit) (%)	74.2	90.1 (0.16)	81.7 (0.10)	72.6 (0.34)	81.7 (0.11)	61.0 (0.27)	72.5 (0.01)	–	–	–	–
	Births attended by skilled health personnel (%)	78.9	92.3 (0.16)	92.0 (0.10)	83.4 (0.34)	85.6 (0.11)	59.2 (0.28)	79.1 (0.01)	–	–	–	–
	Measles immunization coverage (%)	98.1	97.4 (0.17)	99.4 (0.10)	99.1 (0.33)	97.8 (0.10)	97.1 (0.28)	96.7 (0.02)	–	–	–	–
Ghana, 2008			Western	Central	Greater Accra	Volta	Eastern	Ashanti	Brong Ahafo	Northern	Upper East	Upper West
	Demand for family planning satisfied (%)	40.0	32.7 (0.09)	31.6 (0.11)	55.2 (0.15)	45.6 (0.11)	37.9 (0.10)	42.6 (0.20)	45.0 (0.10)	15.7 (0.08)	31.4 (0.05)	43.6 (0.02)
	Antenatal care coverage (at least one visit) (%)	95.4	95.7 (0.09)	92.4 (0.10)	95.7 (0.12)	91.1 (0.09)	96.0 (0.09)	97.3 (0.19)	96.4 (0.10)	95.6 (0.14)	95.7 (0.06)	97.6 (0.03)
	Births attended by skilled health personnel (%)	58.7	61.7 (0.09)	54.0 (0.10)	84.3 (0.12)	53.7 (0.08)	60.8 (0.09)	72.6 (0.19)	65.5 (0.09)	27.2 (0.16)	46.7 (0.05)	46.1 (0.03)
	Measles immunization coverage (%)	90.2	89.7 (0.09)	87.3 (0.10)	92.4 (0.11)	92.0 (0.08)	86.8 (0.10)	93.0 (0.21)	95.7 (0.09)	80.5 (0.14)	96.5 (0.05)	96.7 (0.03)
Zimbabwe, 2010			Manicaland	Mashonaland Central	Mashonaland East	Mashonaland West	Mashonaland North	Mashonaland South	Midlands	Masvingo	Harare/ Chitungwiza	Bulawayo
	Demand for family planning satisfied (%)	82.6	79.9 (0.14)	88.0 (0.11)	85.6 (0.10)	86.6 (0.13)	79.5 (0.04)	64.3 (0.04)	81.0 (0.12)	82.8 (0.10)	82.8 (0.17)	81.8 (0.04)
	Antenatal care coverage (at least one visit) (%)	89.8	86.7 (0.14)	91.8 (0.11)	86.8 (0.10)	87.4 (0.12)	92.9 (0.05)	95.9 (0.05)	91.5 (0.12)	94.1 (0.11)	87.0 (0.16)	92.1 (0.04)
	Births attended by skilled health personnel (%)	66.2	60.5 (0.15)	51.4 (0.11)	59.9 (0.09)	55.0 (0.13)	65.7 (0.05)	71.6 (0.05)	64.6 (0.13)	75.2 (0.11)	83.5 (0.15)	88.4 (0.04)
	Measles immunization coverage (%)	79.1	65.0 (0.17)	81.0 (0.09)	82.0 (0.12)	80.8 (0.10)	91.0 (0.05)	85.4 (0.06)	80.6 (0.12)	77.9 (0.11)	81.1 (0.14)	88.0 (0.05)

### Comparison of 11 summary measures

Table 3 contains 11 summary measures of subnational regional inequality for the four indicators, building on the disaggregated data in Table 2. Higher numerical values indicate more pronounced inequalities. Red and green shading shows the health indicator with the highest and lowest inequality, respectively, for each measure within each study country.

**Table 3:**
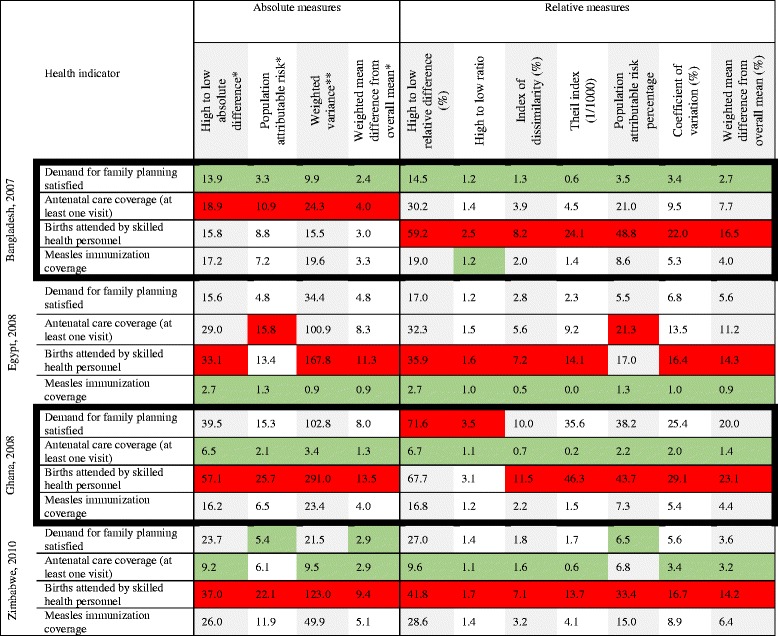
Four reproductive, maternal, newborn, and child health intervention indicators: within-country regional inequality calculated by selected absolute or relative summary measures, Bangladesh, Egypt, Ghana, and Zimbabwe, DHS 2007–2010

In Bangladesh all summary measures indicated lowest inequality in demand for family planning satisfied. Absolute regional inequality was highest for antenatal care, whereas relative inequality was most marked for births attended by skilled health personnel, which had much lower coverage than the other indicators.

For Egypt, all summary measures reported lowest inequality in measles immunization coverage. The two measures of impact (attributable risk), suggested that the antenatal care had the highest degree of inequality, but all other measures suggested that coverage of births attended by skilled health personnel was the most unequal indicator.

In Ghana antenatal care coverage demonstrated the lowest inequality by all measures. Births attended by skilled health personnel tended to show the highest inequality, although relative pairwise measures indicated slightly greater inequality in demand for family planning satisfied.

In Zimbabwe, summary measures tended to report lowest inequality in antenatal care coverage. Exceptions are the two measures of impact (according to these, demand for family planning satisfied had the lowest value), and weighted mean difference from overall mean (which indicated equally low inequality in antenatal care and family planning indicators). Across all measures, the highest inequality was reported for coverage of births attended by skilled health personnel.

In general, indicators with very high national coverage in each country tended to show the smallest magnitude of absolute and relative inequalities across summary measures. Looking across the four countries, skilled birth attendance was the most unequal coverage indicator in all four, whereas the most equitable indicator varied: antenatal care in Ghana and Zimbabwe, family planning in Bangladesh, and measles immunization in Egypt.

### Comparison of four summary measures over time

In this section we compare the performance of four summary measures over time in demand for family planning satisfied: two pairwise measures (difference and ratio), and two measures of variance that take into account all groups (absolute and relative versions of weighted mean difference from overall mean).

**Fig. 1:**
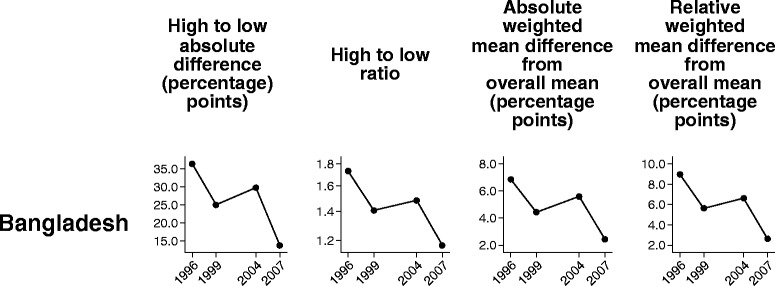
Demand for family planning satisfied in Bangladesh: within-country inequality over time, calculated using four summary measures. Legend: Four summary measures (high to low absolute difference, high to low ratio, absolute weighted mean difference from overall mean, and relative weighted mean difference from overall mean) were calculated to compare their performance in measuring trend over time in within-country inequality for one health indicator (demand for family planning satisfied). Data were sourced from Demographic and Health Surveys conducted in 1996–2007

In Bangladesh all four measures demonstrated similar time trends in subnational regional inequality in the family planning indicator (Fig. 1). Between 1996 and 2007 all summary measures indicated an initial decrease in inequality between the first and second surveys, an approximate leveling off, and then another decrease between the third and fourth surveys.

**Fig. 2:**
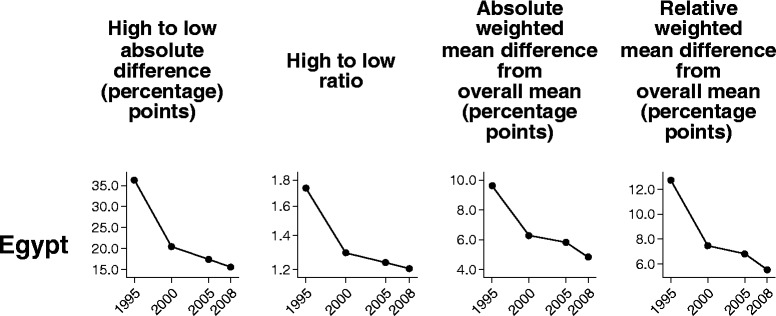
Demand for family planning satisfied in Egypt: within-country inequality over time, calculated using four summary measures. Legend: Four summary measures (high to low absolute difference, high to low ratio, absolute weighted mean difference from overall mean, and relative weighted mean difference from overall mean) were calculated to compare their performance in measuring trend over time in within-country inequality for one health indicator (demand for family planning satisfied). Data were sourced from Demographic and Health Surveys conducted in 1995–2008

Similar time trends were also observed across the four summary measures for family planning in Egypt (Fig. 2). Survey data indicated a decrease in subnational regional inequality between 1995 and 2000, and then a gradual decline through to 2008.

**Fig. 3:**
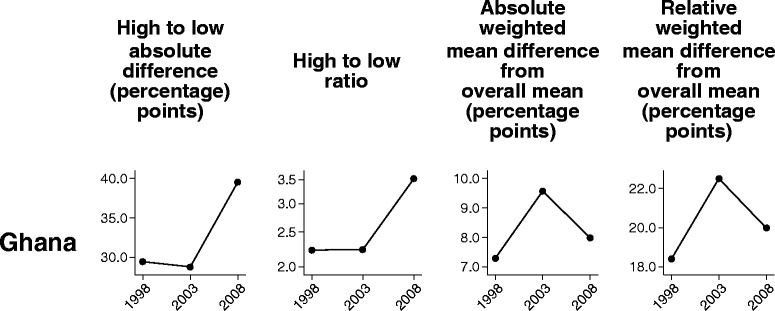
Demand for family planning satisfied in Ghana: within-country inequality over time, calculated using four summary measures. Legend: Four summary measures (high to low absolute difference, high to low ratio, absolute weighted mean difference from overall mean, and relative weighted mean difference from overall mean) were calculated to compare their performance in measuring trend over time in within-country inequality for one health indicator (demand for family planning satisfied). Data were sourced from Demographic and Health Surveys conducted in 1998–2008

**Fig. 4:**
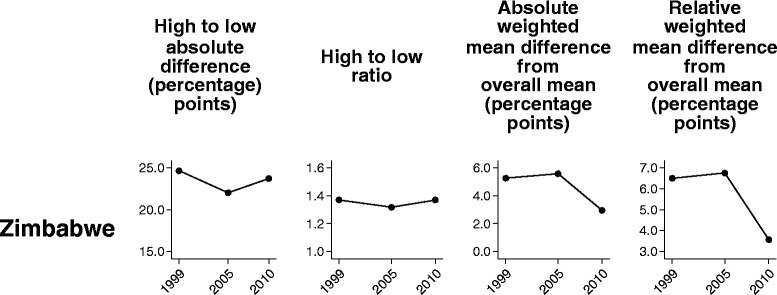
Demand for family planning satisfied in Zimbabwe: within-country inequality over time, calculated using four summary measures. Legend: Four summary measures (high to low absolute difference, high to low ratio, absolute weighted mean difference from overall mean, and relative weighted mean difference from overall mean) were calculated to compare their performance in measuring trend over time in within-country inequality for one health indicator (demand for family planning satisfied). Data were sourced from Demographic and Health Surveys conducted in 1999–2010

In Ghana and Zimbabwe–countries with ten subnational regions–the two pairwise measures suggested a different trend over time than the two measures of variance. In Ghana, whereas pairwise measures showed an increase in inequality between the 2003 and 2008 surveys, the measures of variance indicated a marginal decrease (Fig. 3). In Zimbabwe, pairwise measures showed no change in inequality between 2005 and 2010, and measures of variance suggested a slight decrease (Fig. 4). These discrepancies are linked to the characteristics of the summary measures, which, in the case of pairwise measures, show the inequality between extreme groups and, in the case of measures of variance, take all groups into account.

## Discussion

Drawing on empirical data from four countries, we compared subnational regional inequality in four health services using 11 summary measures. Overall, the 11 different measures are more remarkable for their similarities than for their differences. Although they did not account for all regions, simple pairwise measures tended to support the same conclusions as complex summary measures, that is, by identifying same best and worst coverage indicators in each country and indicating similar time trends.

Given that the 11 different measures produced similar interpretations of the data and led to the same overall conclusions about the situation within each country, how should we select the appropriate measure(s) to report? Consistency is an important overarching criterion, however, there are other factors to consider when measuring and reporting regional inequality.

For the sake of clarity and ease of understanding, reporting simple pairwise measures rather than more complex measures is recommended when both classes of measures arrive at the same conclusion. This is particularly relevant because interpretation of complex measures may be challenging for non-technical audiences. Nevertheless, the option of presenting only pairwise measures needs to be preceded by a review of more complex analyses in order to ensure that these simpler measures accurately reflect the total experience of the country. When applied to a larger number of regions, for example, pairwise measures are more prone to be influenced by outliers. This was illustrated by our comparisons of time trend using pairwise measures and complex measures in countries with ten regions, which revealed some discrepancies. Thus, pairwise comparisons may perform better when applied to a smaller number of subnational regions.

In cases where complex measures of regional inequality are required, mean difference from mean measures offer certain advantages. They are intuitive to interpret, as their outputs either retain the same units as the health indicator or are expressed as percentages. Unlike variance, their calculation does not involve squaring components of the formula. Therefore, these measures resonate with audiences with limited familiarity with statistics. In addition, mean difference from mean measures can be adapted to convey absolute or relative inequality, use weighted or unweighted data, and incorporate various reference groups, such as overall mean or the best region.

Measures of impact–population attributable risk and population attributable risk percentage–may also be intuitively understood by non-technical audiences. They can be particularly powerful, by showing how much can be achieved by eliminating inequality and reaching the level of the reference region. The choice of reference point, however, must be clearly justified. For example, choosing the best-performing region may render the measures sensitive to outliers, which is also a limitation of simple pairwise comparisons. In this case, it would make sense to use a pool of the best performing regions, e.g. those in the top decile of coverage.

Other measures were less intuitive. For example, the Theil index can only demonstrate relative inequality as a weighted measure, with reference to the overall mean. Its output may be difficult to understand as there is no apparent scale. Although the Theil index may be a valid and applicable measure of regional inequality for some contexts [8, 18], it is more difficult to explain and interpret than relative mean difference from mean.

Both measures of absolute inequality and relative inequality should be reported. This is exemplified by the results from Bangladesh. Taking into account the overall level of coverage, the results for skilled birth attendance in Bangladesh was an illustrative example of how an indicator with much lower coverage than the others will perform worse according to relative measures than absolute measures. Although absolute inequality was highest for coverage of antenatal care, relative inequality was highest for coverage of births attended by skilled personnel. Selectively reporting only absolute or relative inequality can affect conclusions about the magnitude and/or trends in inequality, may sway decision making, and reflects a normative judgment about the importance placed on inequality per se [19, 20]. When reported in concert, absolute and relative measures of inequality provide a more complete representation of the situation than either in isolation. In the few cases when authors opt to report only absolute or relative inequality, this should be adequately justified [21].

The selection of geographical units has implications for the magnitude of the resulting inequality, as aggregating subgroups reduces heterogeneity–a so-called “resolution” issue [4, 22]. Thus, it is not possible to directly compare estimates of regional inequality based on variable numbers of units. The resolution issue is a common limitation for all measures of regional inequality, including pairwise measures that compare extreme regions, as well as multiple group measures [22]. In our study, two pairs of countries were selected that had equal numbers of regions, facilitating cross-country comparisons of inequality.

Where applicable, the selection of reference points may also affect monitoring and reporting inequality. The best-performing region at a given point in time may not remain so on a later occasion, or even if it remains it may show particularly rapid or slow progress. This should be borne in mind when using summary measures of inequality that adopt the best region as the reference point.

Normative values and judgments become evident in aspects of measuring and reporting regional inequalities in health. For instance, the use of unweighted versus weighted data signals whether emphasis was placed on measuring inequality between regions themselves (regardless of the population size) or on measures that account for the population size within each region. We note that the data used in these analyses are from household surveys, so the sample size was an important consideration when looking at regional estimates. Taking into account confidence intervals can be useful to indicate the uncertainty around the estimates due to sampling error [2].

## Conclusions

Reporting health inequalities should be transparent and upfront about the judgments that underlie measurement and reporting choices. As stressed above, summary indices should not replace the careful examination of levels and trends within each region and at national level. Our empirical analyses compared indicators with variable levels of national coverage. Reporting on time trends should consider the level of the health indicator at baseline, as there is more room for progress in countries with lower levels of baseline coverage, and also inequalities are likely to be reduced when national coverage approaches 100 %.

Monitoring and reporting health differences across regions has clear practical implications. Unlike the case for wealth-related inequalities–where the poorest quintile, for example, may be spread throughout different regions of a country–geographic inequalities can be used for targeting and deploying interventions to easily-defined disadvantaged subpopulations. Countries such as Brazil [23], Peru, Mexico [24] and Bangladesh (Arifeen S., personal communication) have made use of geographical targeting for reducing overall within country inequalities. Importantly, regional analyses can be applied at any level of geographical unit, such as districts or zones.

Regional inequality also differs from socioeconomic inequality in terms of measurement. While socioeconomic position has an inherently ordered ranking, geographical regions are by nature unordered and cannot be logically ranked. Thus, measures that are employed to quantify socioeconomic inequalities may not be appropriate for the measurement of regional inequality.

The selection of appropriate summary measures to quantify regional inequality entails consideration of the underlying assumptions and value judgments surrounding the use of pairwise versus complex measures, weighted versus unweighted data, and absolute versus relative calculations; where applicable, the choice of reference group and number of geographical units are other important considerations. Nevertheless, our present analyses suggest that a subset of the eleven measures studied are sufficient in most case. We recommend that four measures should be employed when monitoring subnational regional inequality: extreme groups pairwise difference and ratios, and mean differences from mean expressed in absolute and relative scales. When pairwise and complex measures draw the same conclusions about the state of subnational regional inequality, pairwise measures may be sufficient for reporting inequality (unless a more-nuanced assessment is needed). In cases where complex measures are required, mean difference from mean measures can be easily explained and interpreted by non-technical audiences.

## Abbreviations

DHS: demographic and health survey
